# Generation of mouse model of TGFBI-R124C corneal dystrophy using CRISPR/Cas9-mediated homology-directed repair

**DOI:** 10.1038/s41598-020-58876-w

**Published:** 2020-02-06

**Authors:** Kohdai Kitamoto, Yukako Taketani, Wataru Fujii, Aya Inamochi, Tetsuya Toyono, Takashi Miyai, Satoru Yamagami, Masahiko Kuroda, Tomohiko Usui, Yasuo Ouchi

**Affiliations:** 1 Department of Ophthalmology, Graduate School of Medicine, University of Tokyo, Tokyo, Japan; 2 Schepens Eye Research Institute, Boston, MA USA; 3 Graduate School of Agricultural and Life Sciences, University of Tokyo, Tokyo, Japan; 4 Department of Ophthalmology, Nihon University, Tokyo, Japan; 5 Department of Molecular Pathology, Tokyo Medical University, Tokyo, Japan; 6 Department of Ophthalmology, International University of Health and Welfare, Tokyo, Japan; 7 Department of Regenerative Medicine, School of Medicine, Chiba University, Chiba, Japan; 8 Gene Expression Laboratory, Salk Institute for Biological Studies, Ja Jolla, CA, USA

**Keywords:** Corneal diseases, Experimental models of disease

## Abstract

Mutations in transforming growth factor-beta-induced (*TGFBI*) gene cause clinically distinct types of corneal dystrophies. To delineate the mechanisms driving these dystrophies, we focused on the R124C mutation in *TGFBI* that causes lattice corneal dystrophy type1 (LCD1) and generated novel transgenic mice harbouring a single amino acid substitution of arginine 124 with cysteine in TGFBI via ssODN-mediated base-pair substitution using CRISPR/Cas9 technology. Eighty percent of homozygous and 9.1% of heterozygous TGFBI-R124C mice developed a corneal opacity at 40 weeks of age. Hematoxylin and eosin and Masson trichrome staining showed eosinophilic deposits in subepithelial corneal stroma that stained negative for Congo-red. Although amyloid deposition was not observed in TGFBI-R124C mice, irregular amorphous deposits were clearly observed via transmission electron microscopy near the basement membrane. Interestingly, we found that the corneal deposition of TGFBI protein (TGFBIp) was significantly increased in homozygous TGFBI-R124C mice, suggesting a pathogenic role for the mutant protein accumulation. Furthermore, as observed in the LCD1 patients, corneal epithelial wound healing was significantly delayed in TGFBI-R124C mice. In conclusion, our novel mouse model of TGFBI-R124C corneal dystrophy reproduces features of the human disease. This mouse model will help delineate the pathogenic mechanisms of human corneal dystrophy.

## Introduction

Corneal dystrophy is a hereditary disease that causes corneal opacity; 22 types of corneal dystrophy are currently classified according to the International Committee for Classification of Corneal Dystrophies (IC3D)0^1^. Among these, transforming growth factor-beta-induced (TGFBI) corneal dystrophies occur most frequently in East Asian populations^2^. TGFBI corneal dystrophies are caused by a point mutation, or insertion or deletion of some frames, in *TGFBI*, which is located on chromosome 5q31^1,2^. Various mutations in *TGFBI* cause corneal opacities with different phenotypes, such as granular corneal dystrophy (GCD), lattice corneal dystrophy (LCD), Reis-Bücklers corneal dystrophy (RBCD), and Thiel-Behnke corneal dystrophy (TBCD)^3^. There are genotype-phenotype correlations in TGFBI corneal dystrophy; for example, the R124H mutation causes GCD type2 (GCD2), and the R124C mutation causes LCD type1 (LCD1)^2^. Even though we know that the corneal opacities are caused by accumulated TGFBI protein (TGFBIp)^4^, the mechanism or pathophysiology is not yet clearly understood^5^.

Animal models help elucidate the pathophysiology of corneal dystrophies. Previously, Bustamante *et al*. used a lentiviral vector to generate knock-in mice overexpressing the human R555W mutation, which causes GCD type1 (GCD1)^6^. However, R555W mutant mice did not show any corneal phenotype according to this report^6^. *Tgfbi-*knockout mice also failed to show corneal abnormalities even after systemic depletion of *Tgfbi* expression^7^. Recently, Yamazoe *et al*. established an R124H mutant transgenic mouse model showing corneal opacities^8^. However, these mice harbour an artificial human TGFBI mutant gene cassette and do not reflect the complexity of the human disease. Therefore, it is imperative to develop physiologically relevant animal models to decipher the mechanisms driving TGFBI corneal dystrophy.

The popularity of clustered regularly interspaced short palindromic repeats/CRISPR-associated protein 9 (CRISPR/Cas9) gene-editing technology has increased dramatically because it enables rapid and precise genetic manipulation of various cell types^9–11^. Moreover, CRISPR/Cas9 is a powerful one-step strategy for establishing knockout animal models because it allows single-nucleotide conversion via single-stranded donor oligodeoxynucleotide (ssODN) template-mediated homology-directed repair (HDR)^12,13^. Single-nucleotide polymorphism (SNP)-based animal models of human disease are a useful and physiologically relevant approach that reflects point mutations or substitutions in the human genome.

To understand the effect of TGFBI mutation in corneal dystrophy, we generated mutant mice with the Tgfbi R124C mutation, which is the representative mutation causing LCD1, using the CRISPR/Cas9 approach. We then examined how the expression of this phenotype affected corneal integrity and wound healing in this mouse model.

## Results

### Generation of mouse model of TGFBI-R124C corneal dystrophy using ssODN-mediated base-pair substitution introduced via CRISPR/Cas9

**Figure 1:**
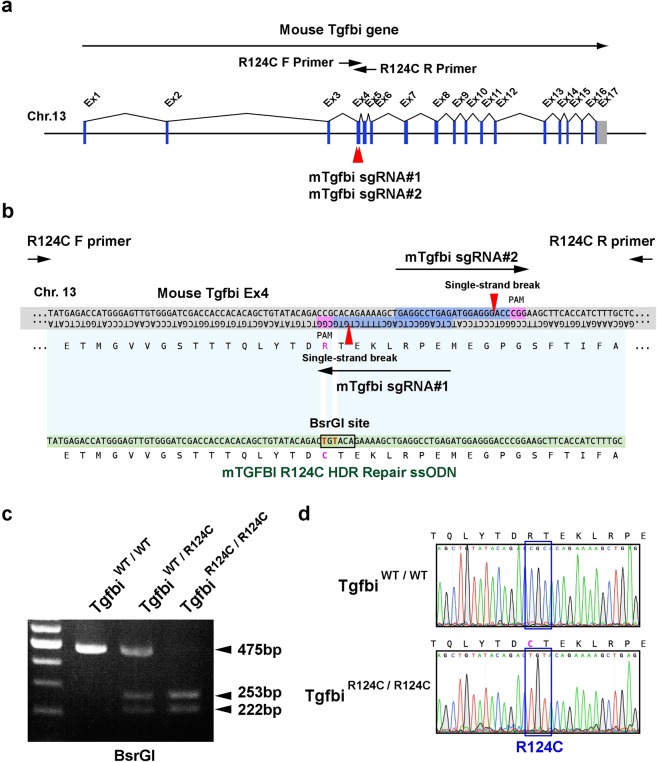
Generation of TGFBI-R124C mouse model via offset-nicking by CRISPR/Cas9 system. (**a**,**b**) Schematic representations of targeting strategy for the generation of TGFBI-R124C mice. Two sgRNAs were designed and *in vitro*-transcribed to target exon 4 in *Tgfbi*. The red arrowhead indicates the expected cleavage site for sgRNAs (**a**). Schematic of sgRNAs and ssODN donor sequences designed to target *Tgfbi* for HDR-mediated knock-in via CRISPR/Cas9 (**b**). The target nucleotide sequence used to induce TGFBI-R124C mutation is indicated in red. The red arrowhead indicates the expected nicking sites for sgRNAs. Tgfbi sgRNA#1 and Tgfbi sgRNA#2 are marked in blue. Protospacer adjacent motifs (PAMs) are highlighted in pink. (**c**) PCR-RFLP genotyping of TGFBI-R124C mutant mice. The image shows an undigested 475 bp fragment in wild-type mice; fragments of 253, 222 and an undigested fragment of 475 bp in heterozygous TGFBI-R124C mice; and fragments of 253 and 222 bp in homozygous TGFBI-R124C. (**d**) Representative sequencing results used to validate the mutation in *Tgfbi* of TGFBI-R124C homozygous mutant mice. In TGFBI-R124C mice, a base substitution (CGC → TGT) is observed.

We used CRISPR/Cas9-induced HDR-mediated nucleotide conversion technology to develop a clinically relevant mouse model of TGFBI corneal dystrophy^14^. We targeted the arginine residue at position 124 of TGFBI; this amino-acid residue is important in the development of TGFBI corneal dystrophy. To generate Tgfbi R124C knock-in mice without inducing off-target cleavage of CRISPR/Cas9, we took advantage of Cas9 nickase (Cas9^D10A^), a mutated Cas9 that makes single-strand breaks, which allows us to generate region targeted knock-in mice without off-target mutations^13,15,16^. Two single-guide RNAs (sgRNAs) were designed to target exon4 of *Tgfbi* (Fig. 1a). To convert arginine 124 into cysteine, we designed a 107 nt ssODN containing the BsrGI restriction enzyme site (Fig. 1b). The BsrGI restriction enzyme site enables easy genotyping of TGFBI-R124C mice by PCR-RFLP analysis, without affecting the amino-acid sequence of the Tgfbi R124C protein. A mixture of Cas9^D10A^ mRNA, sgRNAs, and ssODN was injected into zygotes, as reported previously^13^. Then, two-cell embryos were transferred into pseudopregnant recipient female mice. The genotype of the resultant pups was examined by PCR-RFLP analysis. We PCR amplified 475 bp fragment around the target site and digested the PCR product with BsrGI restriction enzyme. As the PCR product including a cleavage site for BsrGI, PCR-RFLP digestion using BsrGI produced an undigested 475 bp fragment in wild-type mice, and fragments of 253 and 222 bp in homozygotic TGFBI-R124C mice (Fig. 1c). The genotype of the pups was further examined using DNA sequencing, and 5 in 19 pups had the TGFBI-R124C mutant allele. DNA sequencing of Tgfbi^R124C/R124C^ mice confirmed that the mutation had been successfully induced (Fig. 1d). One of the mutant mice was used for breeding, and the descendants were used for the experiments. Both Tgfbi^R124C/R124C^ and Tgfbi^R124C/wt^ mice were viable and fertile, showing normal body weight and life span.

### TGFBI-R124C mice develop corneal opacity with high frequency

**Figure 2:**
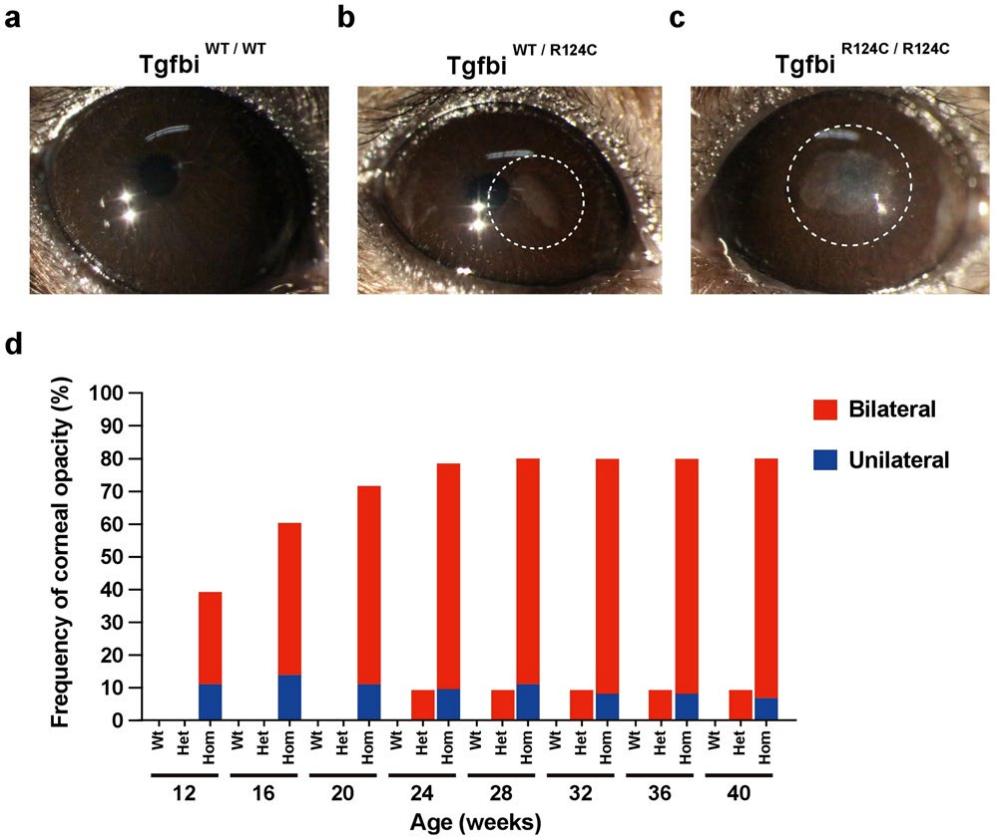
Corneal opacity in TGFBI-R124C mice. (**a–c**) The representative pictures of wild-type at 28 weeks of age mouse and TGFBI-R124C mice eyes at 32 weeks of age. Corneal opacity is not observed in wild-type mice (**a**). Corneal opacity is observed in the centre of the cornea in heterozygous (**b**) and homozygous mice (**c**). Dashed line represents corneal opacity. (**d**) Frequency of corneal opacity by genotype. In Tgfbi^R124C/R124C^ mice, corneal opacity occurred in 78.9% at 24 weeks of age. Then the frequency of corneal opacity gradually increased to 80.3% of mice at 40 weeks of age. Over 91% of the diseased TGFBI-R124C homozygous mice showed bilateral corneal opacity at 40 weeks of age. In eleven Tgfbi^R124C/WT^ mice (Het), bilateral corneal opacity was observed one mouse (9.1%) before 24 weeks of age. No corneal opacity was observed in wild-type mice (Wt).

We used a stereomicroscope to analyse the effects of the TGFBI-R124C mutation on the development of corneal opacity in mice. In TGFBI-R124C homozygous mice, corneal opacity was first observed at 12 weeks of age and tended to gradually worsen until 24 weeks of age. While wild-type littermates (n = 9) did not show any substantial changes in the corneas (Fig. 2a), all corneal opacity was observed in the central cornea of both TGFBI-R124C homozygous and heterozygous mice (Fig. 2b,c). In TGFBI-R124C homozygous mice, corneal opacity was observed in 51 of 71 mice (71.8%) at 20 weeks of age and in 57 mice (80.3%) at 40 weeks of age (Fig. 2d). On the other hand, corneal opacity was not observed in any TGFBI-R124C heterozygous mice below 24 weeks of age, and only 1 of the11 mice (9.1%) developed bilateral corneal opacity until 40 weeks of age (Fig. 2d). Over 71% of the diseased TGFBI-R124C homozygous mice showed bilateral corneal opacity at 12 weeks of age, and the percentage gradually increased to 91% at 40 weeks of age, whereas 5 mice developed unilateral corneal opacity. In summary, total number of the eye showed corneal opacity at 40 weeks of age are 105 (73.9%) and 2 (9.1%) in the TGFBI-R124C homozygous and heterozygous mice, respectively (Table 1).

**Table 1:** Summary of the corneal opacity observed in the TGFBI-R124C mutant mice eye in this study.

Genotypes Total number of eyes analyzed	Tgfbi^WT/WT^ n = 18		Tgfbi^R124C/WT^ n = 22		Tgfbi^R124C/R124C^ n = 142	
Age (weeks)	n	%	n	%	n	%
≦20	0	0	0	0	87	61.3
≦40	0	0	2	9.1	105	73.9

### Abnormal deposits in the subepithelial stroma of TGFBI-R124C mice

**Figure 3:**
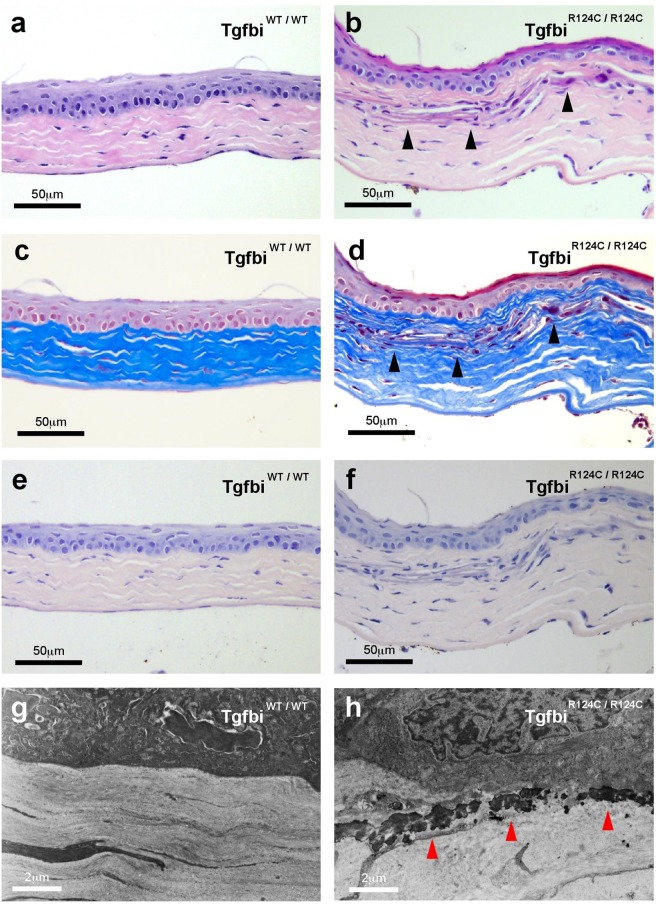
Microscopic examination of the cornea in wild-type and TGFBI-R124C mutant mice. Corneal sections of TGFBI-R124C a homozygous mouse at 22 weeks of age and a wild-type mouse at 24 weeks of age. (**a,b**) H&E staining of corneal tissue shows eosinophilic areas of subepithelial stroma (arrowhead) and no inflammatory cell infiltrate in TGFBI-R124C homozygotes. (**c,d**) Masson trichrome staining showed faint positive staining in the corneal tissue of homozygotic TGFBI-R124C mice (arrowhead). (**e,f**) Congo-red staining of corneal tissue was negative for both homozygotic TGFBI-R124C and wild-type mice. (**g,h**) Transmission electron microscopy (TEM) images of corneal tissues. TEM revealed irregular and amorphous deposits in the subepithelial stroma (red arrowhead). Scale bar: 50 µm in (**a–f**), 2 nm in (**g,h**).

We used histological analysis to evaluate corneal opacity in TGFBI-R124C mice. As demonstrated in Fig. 3a,b, several parts of the subepithelial stroma showed degeneration and fibrosis. No inflammatory cell infiltration was observed. These areas were positive for Masson’s trichrome staining, which indicates an increase in collagen fibres (Fig. 3c,d). All corneal tissues were negative for Congo-red staining (Fig. 3e,f). Transmission electron microscopy (TEM) imaging of the corneas showed pathognomonic, irregular, and amorphous deposits in the subepithelial stroma (Fig. 3g,h).

### TGFBIp deposits in the subepithelial stroma of TGFBI-R124C mice

**Figure 4:**
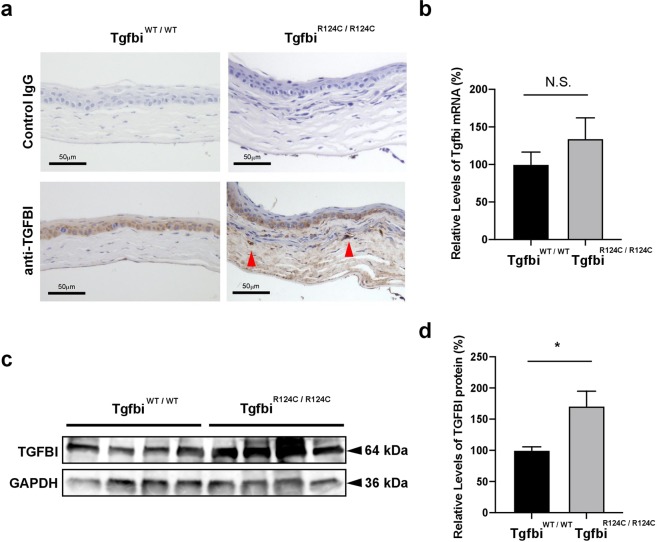
Expression levels of Tgfbi mRNA and protein in the cornea of wild-type and TGFBI-R124C mutant mice. (**a**) Immunohistochemical analysis of TGFBI expression in the cornea of wild-type and homozygotic TGFBI-R124C mice. TGFBI expression was detected in all the corneal layers of homozygotic TGFBI-R124C mice, with particularly strong expression around deposit sites (red arrowhead). No detectable staining was observed in the control sections. Scale bar = 50 µm. (**b**) Real-time RT-PCR analysis of Tgfbi mRNA levels in the corneas of wild-type and TGFBI-R124C mutant mice. The expression level of *Tgfbi* was calculated using the ΔΔCT method. Glyceraldehyde 3-phosphate dehydrogenase (GAPDH) was selected to normalise the gene expression. No significant difference was observed between wild-type and TGFBI-R124C mice (p = 0.31). (**c**)Western blot analysis of Tgfbi protein levels in the corneas of wild-type and TGFBI-R124C mutant mice. d. Comparison of Tgfbi protein expression levels in the corneas of wild-type (n = 4) and TGFBI-R124C homozygotic mice (n = 4). Expression levels of Tgfbi protein were normalised to Gapdh expression. A significant difference was observed between wild-type and TGFBI-R124C mice (p = 0.030).

To assess whether *Tgfbi* mutations result in deposits of TGFBIp in the corneas of TGFBI-R124C mice, we analyzed the expression levels of the *Tgfbi* gene and the TGFBIp protein. First, we performed immunohistochemical analysis to determine the localization of TGFBIp. In the corneas of wild-type mice, TGFBIp immunoreactivity was mainly observed in the corneal epithelium (Fig. 4a). In the corneas of TGFBI-R124C homozygous mice, TGFBIp expression was observed in all the corneal layers (Fig. 4a), whereas no significant staining was observed in control sections.

Expression of Tgfbi mRNA in the corneas of wild-type mice and homozygous mutant mice was examined via real-time reverse transcription-polymerase chain reaction (RT-PCR). Corneal expression of Tgfbi mRNA did not significantly differ between wild-type and TGFBI-R124C mice (Fig. 4b; p = 0.31). Conversely, expression of TGFBIp in the cornea of homozygous mice was significantly increased compared with that of wild-type mice (Fig. 4c,d; p = 0.030, supplementary file).

### TGFBI-R124C mutation affects corneal wound healing

**Figure 5:**
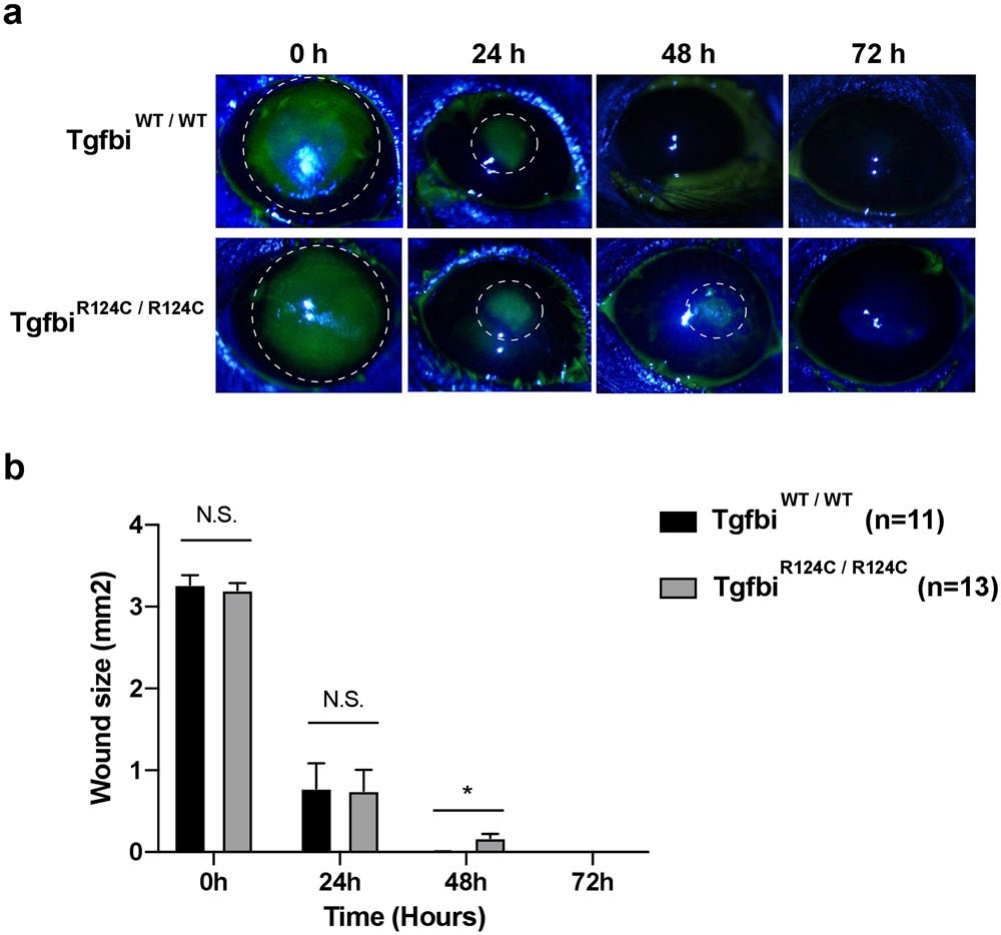
Corneal wound healing in wild-type and TGFBI-R124C mutant mice. (**a**) Representative images of corneal epithelial fluorescein staining at 0, 24, 48, and 72 h, after 2 mm long corneal debridement were induced in wild-type (n = 11) and TGFBI-R124C mutant (n = 13) mice. Dashed line represent stained area. (**b**) Bar graph showing the corneal epithelial healing rates in wild-type and TGFBI-R124C homozygotic mice after induction of 2 mm long corneal debridement. At 48 hours after induction of corneal abrasion, the area bearing an epithelial defect in TGFBI-R124C mice was larger than that in wild-type mice (p = 0.011).

Most patients with R124C LCD present with impaired corneal wound healing^17^; therefore, we examined whether the TGFBI-R124C mutation affected wound healing in TGFBI-R124C homozygous mice. The areas bearing corneal epithelial defects showed no significant differences in wild-type and TGFBI-R124C mice at 24 hours after induction of corneal abrasion. However, at 48 hours after induction of corneal abrasion, the area bearing an epithelial defect in TGFBI-R124C mice was larger than that in wild-type mice (Fig. 5a,b; p = 0.011).

## Discussion

The mechanisms driving the formation of corneal opacity in TGFBI corneal dystrophy are largely unknown^5^. Establishing clinically relevant mouse models of TGFBI corneal dystrophy would be very helpful to understand the mechanisms or pathophysiology underlying corneal opacity. Two animal models of TGFBI corneal dystrophy have been established via introduction of human mutant *TGFBI genes* into mice^6,8^. However, these models were generated by artificial induction of human genetic mutations, and the frequency of corneal opacity observed was insufficient to explain the patient phenotype. Therefore, these models are inadequate for studying the pathogenesis of TGFBI corneal dystrophy.

In this study, we established a mouse model of TGFBI corneal dystrophy using HDR-mediated base substitution introduced via CRISPR/Cas9. To the best of our knowledge, this study is the first to show that a base substitution introduced via CRISPR/Cas9 into *Tgfbi* can induce the development of corneal dystrophy.

In Tgfbi^R124C/R124C^ mice, more than 70% of eyes developed corneal opacity by 40 weeks of age (Table 1). The frequency of corneal opacity in our mutant mice was nearly four times greater than that in previously established mouse models^8^. On the other hand, no corneal opacity was observed in Tgfbi^R124C/WT^ mice up to 20 weeks of age, and the frequency of corneal opacity by 40 weeks was lower than that in Tgfbi^R124C/R124C^ mice. In cases of TGFBI corneal opacity, homozygous patients have earlier onset than heterozygous patients^18^; our finding is consistent with that. Among the mutant mice produced by Yamazoe *et al*., 8% of heterozygous mutant mice were found to have corneal opacity by 40 weeks of age^8^; the same result was obtained for the TGFBI-R124C mice that we studied.

Although corneal opacity was mostly bilateral in the Tgfbi^R124C/R124C^ mice, some mice developed unilateral corneal opacity (Fig. 2d). Among the TGFBI-R124C mice with unilateral corneal opacity, some mice developed bilateral corneal opacity. In cases of TGFBI corneal dystrophy, some cases with unilateral corneal opacity developed corneal opacity in the other eye several years later^19^. It is possible that even in the mice that developed unilateral corneal opacity during our study, bilateral corneal opacity could occur later.

In patients with TGFBI corneal dystrophy (such as lattice and granular dystrophy), deposits and opacities occur in the centre of the cornea^3,20^. Consistent with the patterns observed in human corneal dystrophy, we detected a well-defined mass in the central cornea of the TGFBI-R124C mice (Fig. 2b,c). Corneal deposits in TGFBI-R124C homozygous mice appeared more frequently and developed earlier than in TGFBI-R124C heterozygous mice. Human patients bearing TGFBI-R124H homozygous mutations present with more severe corneal opacity than patients harbouring heterozygous mutations^18,21^. Taken together, our mice model presented very similar symptoms to human corneal dystrophy.

Consistent with patterns of TGFBI corneal degeneration in humans^2^, the deposits in TGFBI-R124C homozygous mice showed eosinophilic staining (Fig. 3b). The deposits caused by the TGFBI-R124C mutation consist of amyloids^3^, which are positively stained by Congo-red^22^. However, positive Congo-red staining was not observed in TGFBI-R124C mice (Fig. 3f). Conversely, the deposits were stained faintly positive by Masson trichrome staining, revealing the presence of hyaline substance (Fig. 3d). These discrepancies may result from differences in degradation of TGFBIp between humans and mice. Corneal opacity manifests as LCD when arginine-124 is replaced with cysteine, as GCD1 when it is replaced with leucine, and as GCD2 when it is replaced with histidine. Korvatska *et al*. speculated that these phenotypic variations in the degradation process are due to this mutation^23^. There are differences in the fragment size of keratoepithelin in the cornea, for each of these diseases^23^.

Conversely, some cases of TGFBI-R124C mutation do not result in amyloid deposits^24,25^. The pathway of TGFBIp degradation is still unclear. Nonetheless, the absence of amyloid deposits in our study may be because TGFBIp in the TGFBI-R124C mice was processed similarly to how it is processed in GCD1 in humans. In humans, GCD1 is caused by the R124L mutation and does not involve amyloid deposits.

The observation periods that we used maybe another reason why we did not observe amyloid deposit formation in TGFBI-R124C mice. In mouse models of amyloid-associated diseases, the formation of amyloid plaques occurs via accumulation of mutant protein and requires a certain length of time. For example, Tg2576 mice, which are used in the well-known model of Alzheimer’s disease, are generated by overexpressing a mutant form of amyloid precursor protein^26^; however, 11–13 months are required to develop sufficient deposits of amyloid plaque^26,27^. Because we did not use artificial overexpression of mutant genes to establish our mouse model of TGFBI corneal dystrophy, our mice likely required more time to form amyloid plaques. Furthermore, because the lifespan of mice is shorter than that of humans, it is possible that there was not sufficient time to form amyloid plaques in our mice.

Under TEM, hyaline in GCD1 or GCD2 appears as unstructured club-shaped or trapezoidal deposits^3^. In TGFBI-R124C mice, hyaline appeared as an unstructured amorphous deposit, unlike the hyaline observed in human TGFBI corneal dystrophy. Although the reason for this discrepancy is unclear, deposits in R124H transgenic mice also presented an amorphous appearance, similar to that in our TGFBI-R124C mice^8^. Different mechanisms may drive hyaline formation in human and mouse corneas.

Corneal deposits in TGFBI corneal dystrophy may be caused by abnormal accumulation of TGFBIp resulting from mutated *TGFBI*^4^. Our results support this notion because sites with aggregations of TGFBIp also showed eosinophilic deposits in our study (Fig. 4a). TGFBIp was expressed at higher levels in the corneas of TGFBI-R124C mice than in those of wild-type mice (Fig. 4d). Nevertheless, the expression levels of Tgfbi mRNA did not differ significantly between wild-type mice and TGFBI-R124C mice (Fig. 4b). Therefore, accumulations of TGFBIp in the corneas of TGFBI-R124C mice may be caused by abnormal TGFBIp degradation and not enhanced the production of *Tgfbi*. Choi *et al*. previously reported that TGFBIp is usually digested via autophagy, but mutant TGFBIp inhibits the fusion of autophagosomes and lysosomes, which results in TGFBIp accumulation^28^. These mechanisms will be examined further in our future studies of TGFBI-R124C mice.

Patients with TGFBI corneal dystrophy often present with recurrent erosion of the corneal epithelium^29^. In LCD involving R124C mutations, regeneration of corneal epithelium after corneal erosion or phototherapeutic keratectomy requires an extended length of time^17,30^. Our results indicate that regeneration of the corneal epithelium after induction of corneal abrasion required more time in TGFBI-R124C mice than in wild-type mice. The domain containing R124 in fascilin1-1 (FAS1-1) is used for binding to integrin^2^. TGFBIp mediates cell adhesion and spreading via integrin^31^. A study using keratocytes has shown that TGFBI is involved in the proliferation, adhesion, and migration of corneal epithelium^32^. Mutated TGFBIp may have influenced the repair of epithelial wounds in our mice. Also, we assume that mutant TGFBIp affected the basement membrane function of epithelial cells because TGFBIp aggregated near the basement membrane in our mouse model. Taken together, our findings suggest some insights into the kinds of mechanisms underlying delayed wound healing in human corneal dystrophy.

In this study, we established the TGFBI-R124C mutant mouse model. The mice showed corneal opacity similar to that observed in human TGFBI corneal dystrophy. The TGFBI-R124C mutant mice might not be reliable as a model of LCD1, given that amyloid deposition was not detected in these mice. Nonetheless, our findings reveal that the corneal opacity was caused by the accumulation of mutant TGFBIp, similar to that observed in TGFBI corneal dystrophy in humans. Epithelial wound healing was delayed in TGFBI-R124C mice compared with wild-type mice. This delayed wound healing in mutant mice was similar to that observed in human patients with R124C LCD. Characterising corneal dystrophy via our TGFBI-R124C mouse model will help to uncover the mechanisms driving the pathogenesis of human TGFBI corneal dystrophies.

## Material and Methods

### Ethics statement

All animal care and experiments involving animals were conducted in full compliance with the ARVO Statement for the Use of Animals in Ophthalmic and Vision Research. The study was approved by the Animal Research Committee of The University of Tokyo.

SgRNA design and plasmid construction

We first designed the sgRNAs at the following loci;

sgRNA target1: 5′-CAGGCCTCAGCTTTTCTGTGCGG [Chr13: 56625270 to 56625292 (GRCm38.p4)]

sgRNA target2: 5′-GAGGCCTGAGATGGAGGGACCCGG [Chr13: 56625285 to 56625308 (GRCm38.p4)].

To construct the plasmid encoding sgRNA target#1 and sgRNA target#2 for *Tgfbi*, the respective oligonucleotides corresponding to the protospacer of each sgRNAs were phosphorylated using T4PNK (Takara Bio Inc., Shiga, Japan), annealed, and cloned into a pGEM-T-Easy-T3-BB-sgRNA plasmid via the BbsI restriction enzyme site, as described previously^33^. The oligonucleotide sequences are as follows:

Tgfbi-sgRNA#1-Forward: AAGGCAGGCCTCAGCTTTTCTGTG

Tgfbi-sgRNA#1-Reverse: AAACCACAGAAAAGCTGAGGCCTG

Tgfbi-sgRNA#2-Forward: AAGGGGAGGCCTGAGATGGAGGGACC

Tgfbi-sgRNA#2-Reverse: AAACGGTCCCTCCATCTCAGGCCTCC

*In-vitro* transcription of sgRNAs and Cas9 nickase mRNA

SgRNAs and Cas9-nickase (Cas9^D10A^) mRNA were prepared as described previously^13^. Briefly, the constructed sgRNA plasmids and pCAG-T3-hCasD10A-pA (Addgene#51638)^13^ plasmid were linearised using the DraI and SphI, restriction enzymes, respectively. SgRNAs and Cas9^D10A^ mRNA were transcribed *in vitro* using T3-RNA polymerase (Promega, CA), according to the manufacturer’s protocol. The template DNAs were digested with 1 U of TURBO DNase (Thermo Fisher Scientific, MA) at 37 °C for 30 min. Then the RNA transcripts were extracted with phenol: chloroform precipitated with absolute ethanol with ammonium acetate, washed, dried, and resuspended in RNase-free water. The RNA solutions were stored at −80 °C until use.

### Establishment of TGFBI-R124C mouse model

In the 124th amino acid of mouse *Tgfbi* exon 4, arginine (CGC) was replaced with cysteine (TGT) by offset-nicking of CRISPR/Cas9 system^13^ with using the two sgRNAs. The ssODN was designed as follows:

5′-TATGAGACCATGGGAGTTGTGGGATCGACCACCACACAGCTGTATACAGATGTACAGAAAAGCTGAGGCCTGAGATGG AGGGACCCGGAAGCTTCACCATCTTTGC-3′

Mutant mice were generated as reported previously^13^. Briefly, 100 ng/μl Cas9^D10A^ mRNA, 10 ng/μl of each sgRNAs, and 200 ng/μl ssODN were injected into the zygotes of C57BL/6NCr mice. The two-cell embryos were transferred into the oviductal ampullae of 8-week-old female mice from the Institute of Cancer Research (ICR) mated with vasectomized ICR males.

After birth, genomic DNA was extracted from the tail tip using Nucleospin Kit (TaKaRa Bio Inc., Shiga, Japan). The obtained sample was amplified via the following primers using a KOD FX kit (Toyobo Co., Ltd., Osaka, Japan):

R124C-Forward: 5′-CATCTGACTCCTGCGGTTCC-3′

R124C-Reverse: 5′-CTTTCACTTTCCTTGGGGCTG-3′

PCR products were then sequenced to ensure that the mutation had been introduced.

### RFLP assay

RFLP assay was performed to assess the genotype of mice used in this study. DNA was extracted from the mice, and the mutation site was amplified via PCR as described in the previous section. The obtained PCR products were treated with the restriction enzyme BsrGI (New England Biolab, Ipswich, MA) and electrophoresed on a 2% agarose gel.

### Determination of corneal opacity

Eighteen eyes from 9 wild-type mice, 22 eyes from 11 heterozygous mice, and 142 eyes from 71 homozygous mice were examined using a slit lamp. The examination was performed under general anaesthesia induced via intramuscular injection of a xylazine (5 mg/ml) and ketamine (50 mg/ml) mixture. Examinations were conducted every month from the age of 12 weeks to 40 weeks. Mice were divided into those with bilateral or unilateral corneal opacity. The age of the mice and the presence of corneal opacity was recorded.

### Histology

Eyes were removed from 22-week-old mice under general anaesthesia induced as mentioned previously. The eyes were fixed in 4% paraformaldehyde, after which the corneal buttons were excised, subjected to standard processing, embedded in paraffin, and sectioned at 5–7 μm. These sections were then deparaffinised using xylene, dehydrated using graded ethanol, and stained using H&E, Congo-red to detect the presence of amyloids and Masson’s trichrome to detect hyaline, as per the manufacturers’ instructions.

The deparaffinised sections were also evaluated using immunohistochemistry. The sections were washed with PBS, blocked with 5% skim milk for 30 min at room temperature, and incubated with a rabbit anti-TGFBI antibody (Proteintech Group Inc., Rosemont, IL;#10188-1-AP, 1:1000) for 60 min at room temperature. Adjacent sections were processed without primary antibody used as negative controls. Peroxidase-labelled anti-rabbit polyclonal antibody (Nichirei Biosciences Inc., Tokyo, Japan, # 714341) was used as the secondary antibody and the sections were incubated for 30 min at room temperature. Immunoreactivity was visualized using the Dako Liquid DAB + Substrate Chromogen System (Agilent Technologies, Inc., Santa Clara, CA, # K3467).

### Transmission electron microscopy

Enucleated eyeballs were fixed using 4% paraformaldehyde and 2.5% glutaraldehyde and then embedded in epoxy resin. Ultrathin sections were double-stained with 2.5% gadolinium acetate and lead citrate and examined using an electron microscope (JEM1200EX II, JEOL Ltd., Tokyo, Japan).

### Real-time reverse transcription PCR

Total RNA, obtained from mouse corneas, was processed using ISOGEN (Nippon Gene, Toyama, Japan). cDNA was obtained using PrimeScript RT Master Mix (TaKaRa Bio Inc.). Real-time RT-PCR was performed using SYBR Premix Ex Taq II (Tli RNaseH Plus, TaKaRa Bio Inc.). Thermal cycling conditions for RT-PCR were as follows: 40 cycles of 95 °C for 5 s and 60 °C for 30 s. The expression level of the target gene was calculated using the ΔΔCT method. Glyceraldehyde 3-phosphate dehydrogenase (GAPDH) was selected to normalize the gene expression. Primers used for mouse Tgfbi and Gapdh are shown below.

Tgfbi-mRNA-Forward: 5′-AGAGGAAGATCTGCGGCAAG-3′

Tgfbi-mRNA-Reverse: 5′-TCTCGGCAGGGATCTTCTCA-3′

Gapdh-mRNA-Forward: 5′-CACATTGGGGGTAGGAACAC-3′

Gapdh-mRNA-Reverse: 5′-AACTTTGGCATTGTGGAAGG-3′

### Western blot analysis

Corneas were ground in liquid nitrogen and then homogenised in 90 μl radioimmunoprecipitation assay (RIPA) buffer on ice. Homogenised samples were agitated for 2 h and centrifuged for 15 min at 4 °C at 14,000 × *g*. Then, 10 μg protein was loaded per lane, separated on a 10% acrylamide gel (Bio-Rad, Richmond, CA) via sodium dodecyl sulfate-polyacrylamide gel electrophoresis (SDS-PAGE), and transferred to polyvinylidene difluoride (PVDF) membranes using the Trans-Blot Turbo System (Bio-Rad). Membranes were blocked using Blocking One solution (Nacalai Tesque, Inc., Kyoto, Japan) and incubated with a rabbit anti-TGFBI antibody (1:500, Protein Tech) or mouse anti-GAPDH antibody (1:1000, Abcam, Cambridge, UK, # ab8245) overnight at 4 °C. ﻿After washing with TBS-T, the membranes were incubated with HRP-conjugated secondary antibody (Goat anti-rabbit IgG, 1:5000,# 32460 or Goat anti-mouse IgG, 1:5000,# 32430) at room temperature for 3 h. Immunodetection was performed via chemiluminescence using Super Signal West Femto Maximum Sensitivity Substrate (Thermo Fisher Scientific, IL) and ImageQuant LAS 4000 mini (Fujifilm, Tokyo, Japan). The expression levels of TGFBIp were evaluated with respect to that of GAPDH.

### Corneal wound healing model

Corneal epithelial debridement was performed using a previously reported method with several modifications^34^. A circle of filter paper (diameter 2 mm) was soaked with 70% EtOH and placed onto the central cornea for 30 s; then, the corneal epithelium was debrided with a #11 scalpel. The cornea was evaluated using fluorescein staining at 0, 24, 48, and 72 h after debridement. The wound area was calculated using ImageJ (https://imagej.nih.gov/ij/).

### Statistical analysis

Comparisons between groups were performed using the Wilcoxon rank-sum test. P < 0.05 was considered statistically significant. All statistical analyses were performed using JMP Pro 14 (SAS Institute, Cary, NC).

## Supplementary information

Supplementary Information.

## Data Availability

The datasets analyzed during the current study available from the corresponding author on reasonable request.
