# Supplementary figures and images for: Generation of mouse model of TGFBI-R124C corneal dystrophy using CRISPR/Cas9-mediated homology-directed repair
Source: Sci Rep. 2020 Feb 6;10:2000. doi: 10.1038/s41598-020-58876-w (PMC7005300; doi:10.1038/s41598-020-58876-w)

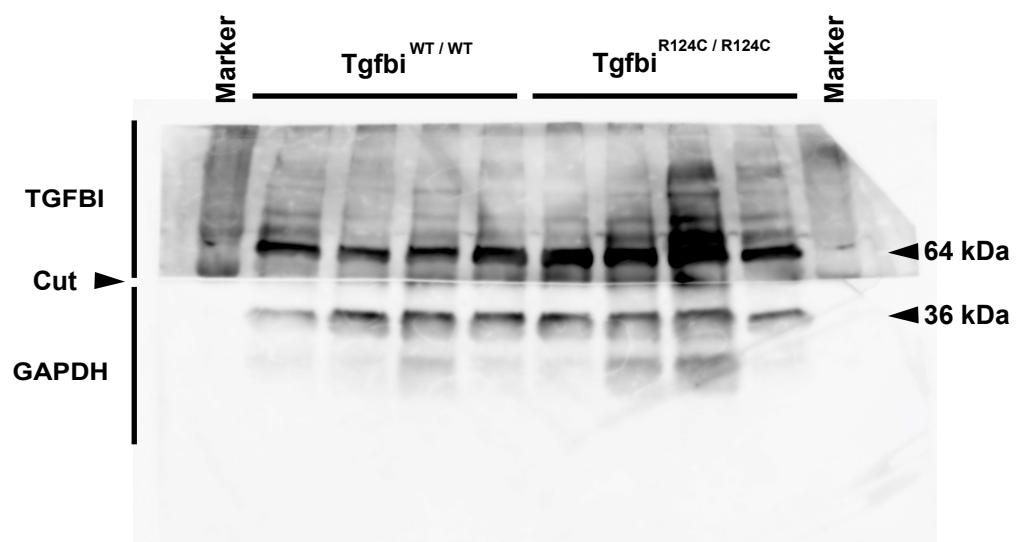

Supplement: Supplementary file 1 — Supplementary Information. [file 41598_2020_58876_MOESM1_ESM.pdf]
